# A Rare Case Report of the Successful Management of Perforated Sigmoid Volvulus in a Pregnant Woman With Massive Pneumoperitoneum: First Case in Yemen

**DOI:** 10.7759/cureus.72289

**Published:** 2024-10-24

**Authors:** Mohammed A Saghir, Hebat Allah N Fadhl, Sarah Mohammed, Abdurazaq Saeed, Mariam Alsakkaf

**Affiliations:** 1 Postgraduate, Faculty of Medicine, Cairo University, Cairo, EGY; 2 Graduate College, University of Bahri, Khartoum, SDN; 3 Medical Education, Faculty of Medicine and Health Sciences, Aden University, Aden, YEM; 4 Surgery, Faculty of Medicine and Health Science, Omdurman Islamic University, Omdurman, SSD; 5 General Surgery, Aden German Hospital, Aden, YEM; 6 Surgery, Aden German Hospital, Aden, YEM

**Keywords:** intestinal obstruction, peritonitis, pregnancy, sigmoid volovlus, yemen

## Abstract

Sigmoid volvulus during pregnancy is an extremely rare condition that presents as intestinal obstruction and can lead to severe complications for both the mother and fetus if not promptly diagnosed and treated. To our knowledge, this is the first documented case in Yemen. We report a case of a 39-year-old pregnant woman at 32 weeks of gestation who presented with acute abdominal pain, constipation, and signs of peritonitis. Diagnostic imaging was limited due to the pregnancy, but an ultrasound revealed fluid collection in the pelvis and massive pneumoperitoneum, suggesting a rupture of the hollow viscus. An emergency exploratory laparotomy confirmed a perforated sigmoid volvulus with necrosis of the sigmoid wall; as a result, we resected a nonviable colon and performed Hartmann’s procedure. After five days, we discharged the patient in excellent condition, and the fetus was monitored as healthy. Diagnosis of sigmoid volvulus during pregnancy is challenging due to the overlap of symptoms with normal pregnancy. Prompt surgical intervention is crucial to prevent maternal and fetal complications. This case highlights the importance of a multidisciplinary approach involving general surgeons, obstetricians, and neonatologists in managing this rare condition. Early diagnosis and treatment are essential for improving maternal and fetal outcomes in cases of sigmoid volvulus during pregnancy.

## Introduction

Intestinal obstruction is a surgical emergency condition that occurs due to the failure of forward movement of gastrointestinal contents, which can occur as a result of either a dynamic (occlusion of the intestinal lumen) or an adynamic (absent or disordered peristalsis) [1]. Volvulus is a Latin word that means twisting. Colonic volvulus refers to the rotation of a portion of the colon around its mesentery. Colonic volvulus ranks as the third most common cause of colonic obstruction worldwide [2]. Clinical presentations are unspecific, and diagnosis relies on high clinical suspicion. Sigmoid volvulus often manifests acutely as abdominal pain, distention, and vomiting, while its chronic form often presents insidiously with vague signs and symptoms at diagnosis [3].

Intestinal obstruction, or sigmoid volvulus, during pregnancy is an extremely rare event [4]. This disease generally presents as mechanical bowel obstruction, and the physiological symptoms of pregnancy may cloud the clinical picture [5]. Laboratory findings are not sufficient in situations where X-rays and CT scans are generally avoided to avoid risk. Ultrasonography is helpful in assessing fetal development and can diagnose intestinal obstruction in experienced hands [5]. Ultrasonography is useful for assessing fetal development and can diagnose intestinal obstruction in experienced hands. Ultrasonography can detect signs of intestinal obstruction, such as dilated loops, abnormal peristalsis, parietal and valvulae conniventes alterations, and the presence of free extraluminal fluid [6]. If not diagnosed and treated early, sigmoid volvulus can cause maternal complications such as perforation, peritonitis, sepsis, and death. Fetal complications include preterm delivery, intrauterine death, and neonatal sepsis [7]. The most common causes of intestinal obstruction during pregnancy include adhesion, volvulus of the intestine, carcinoma, herniation, and appendicitis [8]. Difficult presentation and delayed diagnosis are responsible for the high morbidity and mortality in this situation.

In this report, we present the case of a pregnant woman diagnosed with sigmoid volvulus with massive pneumoperitoneum and peritonitis who was treated with an open diagnostic laparotomy and underwent resection of the sigmoid colon and stoma formation.

## Case presentation

**Figure 1:**
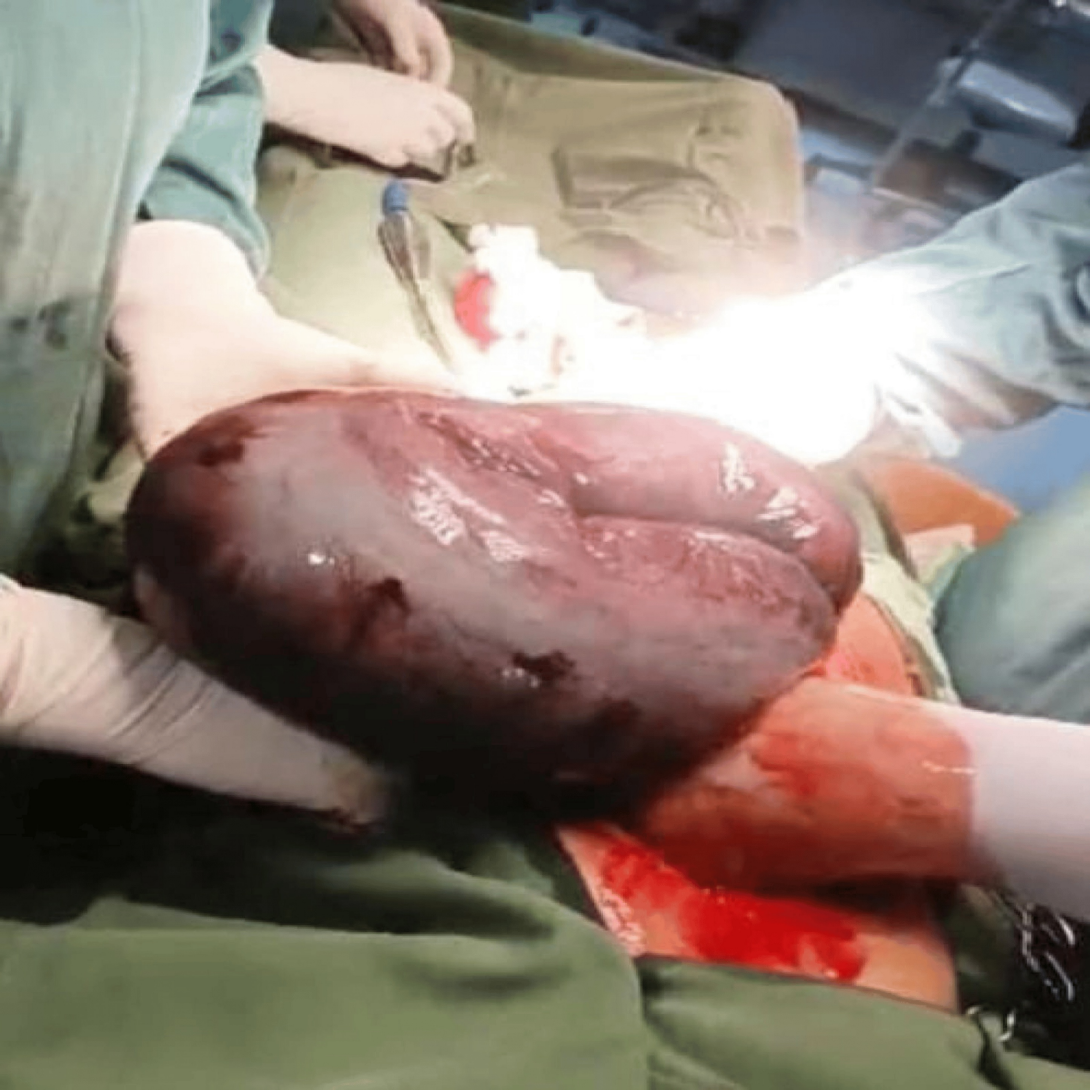
Intraoperative assessment showing a perforated sigmoid volvulus with necrosis of the sigmoid wall

**Figure 2:**
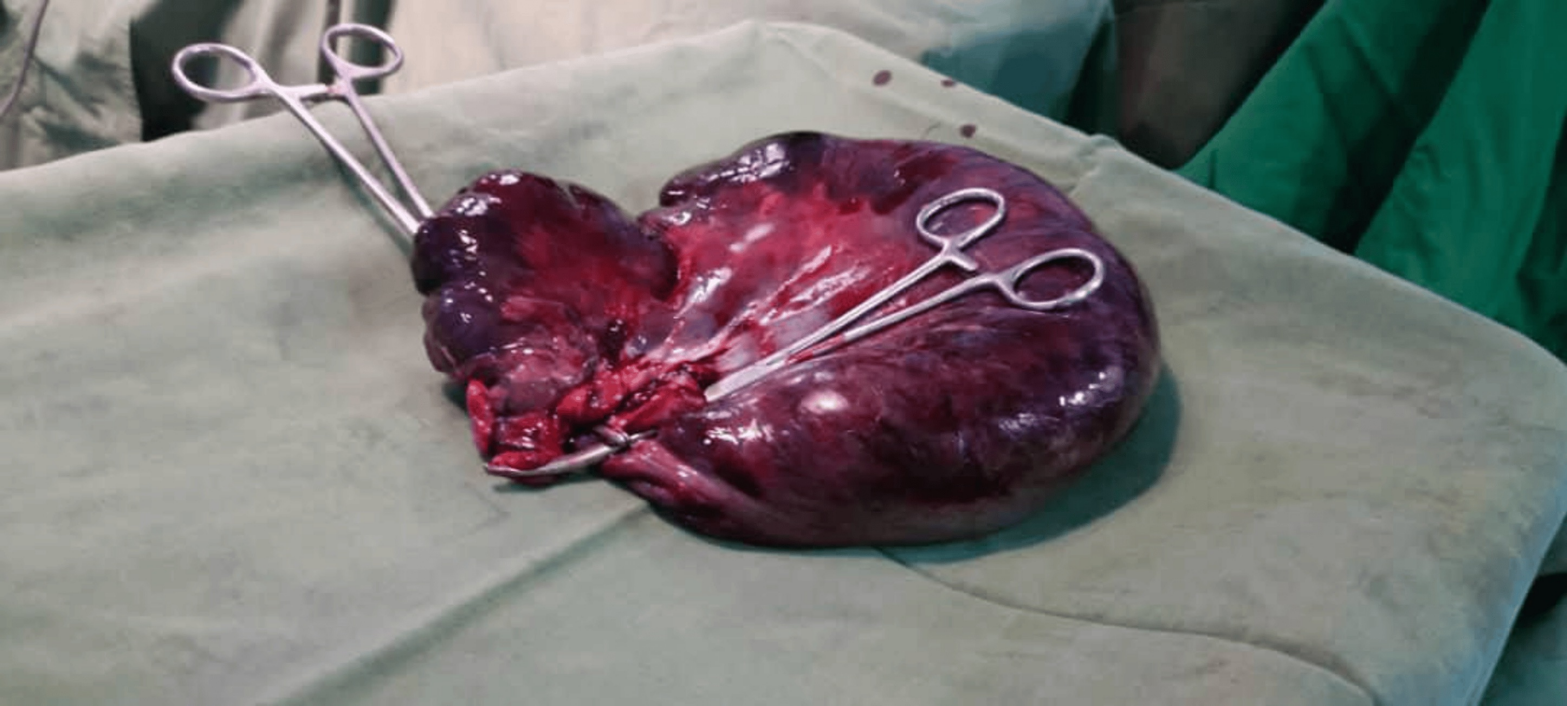
Resected nonviable part of the colon

A 39-year-old pregnant woman, para 1 gravida 3, at 32 weeks of gestation, presented to the emergency department of Aden German International Hospital, Aden, Yemen, on May 22, 2024. She complained of severe acute abdominal pain. The pain gradually increased with time, starting two days prior to constipation associated with tachycardia (pulse rate was 150 bpm), indicating dehydration. The pain was colicky in nature and was not associated with nausea, vomiting, fever, or bleeding per rectum. She passed flatus 27 hours prior to presentation to the hospital, after which she developed absolute constipation for two days. The patient had no relevant medical history. On general examination, the patient appeared ill, tachypneic, and dehydrated. Abdominal examination revealed a distended generalized tender abdomen with hypokinetic bowel sounds showing signs of peritonitis; on digital rectal examination, the rectum was loaded with feces. The blood investigations showed slightly elevated white blood cells (11,800/mm^3^), mainly neutrophils (78%), platelet count (230,000/mm^3^), and a low hemoglobin level of 9.7 g/dL. The renal function test was slightly elevated (creatine was 270 µmol/L), with normal electrolytes: sodium (140 mmol/L), potassium (4.00 mmol/L), and chloride (101 mmol/L) (Table 1). Although a shot of an erect abdominal X-ray may be safe, we avoid X-ray radiography and CT scan images. Ultrasonography revealed fluid collection in the pelvis with massive pneumoperitoneum, which was suspected to be caused by rupture of the hollow viscus due to ischemia and necrosis (complicated intestinal obstruction). After resuscitation, we immediately transferred the patient to the operating room for an emergency exploratory laparotomy. The intraoperative diagnosis revealed a perforated sigmoid volvulus, along with sigmoid wall necrosis (Figure 1), with a moderately unpleasant odor, a dark color-free fluid collection, and multiple uncomplicated hydatid cysts in the right hepatic lobe. We resected the nonviable part of the colon and performed Hartmann’s procedure (Figure 2). Obstetrics and gynecology consultants monitored the patient closely. After five days of good condition, the patient was discharged for clinic follow-up. She had a normal vaginal delivery at the hospital and planned reanastomoses and colostomy closure. 

**Table 1:** Investigation findings

Investigation	Findings	Reference range
Hemoglobin	9.7 g/dL	12.0-16.0 g/dL
White blood cells (WBC)	11,800/mm^3^	4500-11,000/mm^3^
Neutrophils	78%	55-70%
Platelets	230,000/mm^3^	150,000-400,000/mm^3^
Creatine	270 µmol/L	53-97.2 µmol/L
Sodium	140 mmol/L	136-145 mmol/L
Potassium	4.00 mmol/L	3.5-5.2 mmol/L
Chloride	101 mmol/L	96-106 mmol/L

## Discussion

In 1830, Houston was the first to report intestinal obstruction during pregnancy. The incidence is one in 66,431 deliveries [9]. Intestinal obstruction and considerable impact on bowel function can result from various factors, including but not limited to congenital malformations, adhesions, volvulus, intussusceptions, colonic tumors or cancer, appendicitis, and hernias [9]. Constipation, nausea, and bilious vomiting or bile-stained vomiting can develop gradually from colicky abdominal discomfort, a common indication of intestinal obstruction.

The severity of the symptoms can vary depending on the underlying cause of the intestinal obstruction, which could be adhesions, hernias, or tumors [10]. The mechanism that causes sigmoid volvulus during pregnancy is the displacement of a mobile sigmoid colon by an enlarging uterus, especially during the third trimester [11]. This causes the sigmoid colon to leave the pelvis and twist around its point and mesocolon.

Rarely, in late presentation of patients with complete intestinal obstruction, they may present with fever, dehydration, absence of bowel sound, peritonitis, and leukocytosis [12]. Because nausea, vomiting, and abdominal distension are common pregnancy symptoms, diagnosing sigmoid volvulus during pregnancy can be difficult. Subsequently, when a pregnant woman exhibits clinical symptoms of distention, extreme constipation, and stomach pain, a diagnosis should be considered [9].

High white blood cells can be a useful sign, but in the first phase of the disease, they can be normal or slightly elevated [13]. Using radiological investigations can be useful to establish the diagnosis, but many clinicians are restricting their use due to concerns regarding fetal complications. Radiation exposure may lead to chromosomal abnormalities, DNA mutations, and an increased risk of hematologic malignancies [5].

Patient management should include fluids, electrolyte balance correction, antibiotics, and nasogastric decompression. Intervention by an obstetrician should strictly depend on the condition of the fetus. Before terminating the pregnancy, we should administer steroids to enhance fetal lung maturity [14].

Delays in diagnosing and treating sigmoid volvulus can significantly impact the health of both the mother and her fetus, potentially leading to multiple organ failure, hypovolemic shock, septic shock, bowel infarction, and intestinal necrosis [15].

In pregnancy, the prognosis for sigmoid volvulus is poor [9,15]. This case presentation demonstrates the importance of early detection and appropriate care of sigmoid volvulus perforation in preventing maternal mortality.

## Conclusions

Pregnancy-related sigmoid volvulus is a very uncommon disorder that needs multidisciplinary treatment by general surgeons, obstetricians, and neonatologists. Early diagnosis and treatment are critical to ensuring optimal well-being for the mother and fetus. Despite peritonitis symptoms, blood testing may be normal, so a clinical examination and exploratory laparotomy confirm the diagnosis.
